# Endoscopic Findings of 100 Early-Stage Esophageal Cancers

**DOI:** 10.1155/DTE.1.83

**Published:** 1994

**Authors:** Ph. Monnier, Ch. Fontolliet, J. B. Ollyo

**Affiliations:** 1 Otolaryngology Head & Neck Surgery CH-1011CHUV Lausanne Switzerland; 2 Institute of Pathology UNIL Lausanne Switzerland; 3 Gastroenterology Lausanne Switzerland

## Abstract

The morphologic analysis of 100 early squamous cell carcinomas of the esophagus has shown that the barely visible or invisible forms (erythroplakias and occult forms) are predominant. This explains the poor yield of upper gastrointestinal (GI) endoscopies in detecting early cancers, at least in Western countries. Leucoerythroplakias correspond to the most advanced form of early cancers (submucosal invasion in approximately 38% of cases). Pure erythroplakias and occult forms correspond to in situ or intramucosal cancers in over 90% of the cases.

Accurate endoscopic staging is possible using morphologic criteria, superficial spread, and rigidity of the wall as parameters. In a prospective study, we show that the degree of accuracy of this staging system reaches 95% for an experienced endoscopist.

T_1a_N_0_ cancers can benefit from an endoscopic treatment (mucosectomy or photodynamic therapy), because the risk of lymph node metastasis is low (6%). In T_1b_N_0_ cancers, the best treatment option is an esophageal resection with extensive mediastinal lymph node dissection for good surgical candidates; PDT combined with adjuvant radiotherapy is a reasonable option for inoperable patients.


*Diagnostic and Therapeutic Endoscopy*, Vol. 1, pp. 83–92
Reprints available directly from the publisher
Photocopying permitted by license only

© 1994 Harwood Academic Publishers GmbH
Printed in Malaysia

### Endoscopic Findings of 100 Early-Stage Esophageal Cancers

Ph. MONNIER*, Ch. FONTOLLIET† and J. B. OLLYO‡

**Otolaryngology, Head & Neck Surgery, Lausanne, Switzerland*
*†Institute of Pathology, UNIL, Lausanne, Switzerland*
*‡Gastroenterology, Lausanne, Switzerland*

*(Received January 27, 1994; in final form, April 4, 1994)*

The morphologic analysis of 100 early squamous cell carcinomas of the esophagus has shown that the barely visible or invisible forms (erythroplakias and occult forms) are predominant. This explains the poor yield of upper gastrointestinal (GI) endoscopies in detecting early cancers, at least in Western countries. Leucoerythroplakias correspond to the most advanced form of early cancers (submucosal invasion in approximately 38% of cases). Pure erythroplakias and occult forms correspond to in situ or intramucosal cancers in over 90% of the cases.

Accurate endoscopic staging is possible using morphologic criteria, superficial spread, and rigidity of the wall as parameters. In a prospective study, we show that the degree of accuracy of this staging system reaches 95% for an experienced endoscopist.

$T_{1a}N_0$ cancers can benefit from an endoscopic treatment (mucosectomy or photodynamic therapy), because the risk of lymph node metastasis is low (6%). In $T_{1b}N_0$ cancers, the best treatment option is an esophageal resection with extensive mediastinal lymph node dissection for good surgical candidates; PDT combined with adjuvant radiotherapy is a reasonable option for inoperable patients.

**KEY WORDS:** esophagus, early cancer, endoscopy, treatment, photodynamic therapy

### INTRODUCTION

Compared with that in China, Iran, and other Eastern countries, the incidence of esophageal carcinoma is much lower in Europe (< 10/100,000) (Siewert *et al.*, 1977). A systematic screening with sponge or balloon cytology is therefore unadvisable. The only exception to this rule applies to patients with head and neck cancer, up to 97% of whom consumed tobacco and alcohol. For other groups of patients, endoscopy remains practically the only chance to diagnose an early asymptomatic esophageal carcinoma. This endoscopic screening should be integrated in the routine daily work of gastroenterologists and head and neck surgeons. This is why endoscopists should be well aware of the different morphologic patterns of early squamous cell carcinomas of the esophagus (Monnier *et al.*, 1981).

### MATERIAL AND METHODS

Our experience has been gathered from a systematic endoscopic and histopathologic study of 100 early squamous cell carcinomas of the esophagus over a 15-year period (1977–1992). Most of these lesions were diagnosed in patients during endoscopy before or after treatment for head and neck cancer, 48 being synchronous and 34 metachronous early second primaries. In only 13 cases did the endoscopist detect the early cancer as an isolated lesion.

### Technical Details of the Endoscopic Examination

In head and neck cancer patients, the notion of common risk factors (tobacco and alcohol consumption) is coupled with the tendency to develop multifocal lesions. This implies the need not only for endoscopy of the esophagus, but for that of the mouth, pharynx, and tracheobronchial tree.
The basic requirements of this pretherapy endoscopic screening are:

1. high expertise of the endoscopist;
2. exploration of the whole mucosal surface exposed to carcinogens, with special attention to high-risk regions;
3. high quality optical instruments (Hopkins system) and efficient methods of histologic sampling; and
4. capability of detecting early lesions in their inframacroscopic stage (epithelial dysplasia, carcinoma in situ, and microinvasive carcinoma) with the use of vital staining, for example.

Address for correspondence: Dr P. H. Monnier, Otolaryngology, Head & Neck Surgery, CH - 1011 CHUV Lausanne, Switzerland. Phone: 41.21.314.46.40, Fax: 41.21.314.46.43

This oncologically oriented endoscopy calls for a various number of special instruments that provide optimal exploration at all levels of the upper aerodigestive tract. Practically speaking, the patient arrives on an out-patient basis in the morning, fasts, and leaves the clinic in the early afternoon. Examination of the oral cavity and pharynx is carried out using a surgical microscope before and after Toluidine blue gargle. This vital staining procedure, accomplished in less than five minutes, is done in three steps. (1) washing or multiple swabbing with 1% acetic acid (mycolysis); (2) 1-minute washing or swabbing with 1% Toluidine blue (staining); and (3), thorough rinsing with 1% acetic acid (decolorization).

This method aids in directing biopsy sites and in determining the exact extent of a suspicious cancerous lesion. It also facilitates screening of early second primary cancers devoid of macroscopic signs (epithelial dysplasia, carcinoma in situ). The limits of the method must, however, be taken into consideration (several false-positives and one false-negative) (Table 1).

For the pharyngolarynx, the surgical microscope and laryngeal mirror or the Hopkins 90° angle optics (Von Stuckrad indirect laryngoscope) are of considerable help, because they provide a detailed dynamic examination of the high-risk zones with the use of vital staining.

The patient then undergoes general anesthesia. Bronchoscopy is performed first, using both a rigid bronchoscope and a fiberscope. Because the two instruments are complementary, it is possible to perform an optimal quality tracheobronchoscopic examination that offers outstanding optical quality and instrumentation, possibilities for examining the larger bronchi using the rigid bronchoscope, peripheral examination, and biopsy of lesions located distal to the segmentary bronchi using the fiberscope.

**Table 1** False positive and negative reactions to Toluidine blue staining

| *Reaction* | *Explanation* |
|---|---|
| FALSE POSITIVE | |
| Mucosa } erosions, fissures, ulcerations | Artifactual penetration of dye into the mucosa |
| Chronic inflammation | Increased cellularity<br>Increased number of nuclei<br>Frequent erosion |
| Benign tumors | Mechanical retention of dye |
| Ciliated pseudostratified epithelium | Goblet cells (mucus)<br>Affinity of Toluidine blue for mucopolysaccharides |
| FALSE NEGATIVE | |
| Severe dysplasia with parakeratosis | Keratosis prevents the penetration of dye into the epithelium |
| Submucosal extension of tumor | Lack of submucosal penetration of dye |

Note.—With practice, the number of false negatives can be reduced. The esophagus is ideal for vital staining, especially in the presence of early lesions, which turn royal blue in most cases.

If a suspicious lesion is detected in the pharyngolaryngeal region, direct examination is performed with a surgical microscope and a Toluidine blue test. However, the anatomic configuration of this region with its folds and recesses increases the risk of staining artifacts.

Finally, hypopharyngoesophagoscopy is performed using a rigid esophagoscope equipped with Hopkins optics; Toluidine blue is applied systematically to every suspicious lesion. The rigid instrument seems essential for this exploration, because it provides optimal spreading of the mucosa in the recesses of the pharyngolaryngeal region where saliva tends to stagnate, constituting high-risk cancer zones as well as of the sphicter regions. To be completely reliable, the Toluidine blue staining procedure (similar to that performed in the oral cavity) must be done in three steps. Owing to the superior instrumentation possibilities using the rigid endoscope, staining is carried out under optimal conditions, whereas with the gastrofiberscope, the technical difficulties introduce serious problems in the interpretation of a possible lesion such as the impossibility of achieving mechanical decoloration using a cotton swab soaked in acetic acid. If one wishes to use a gastrofiberscope, the Lugol iodine solution is preferable, because it is easier to use. The Lugol iodine solution is a negative tumor marker that does not stain epithelial dysplasias and carcinomas in situ, because these precancerous lesions are devoid of the glycogen present in normal squamous cell epithelium (Mori *et al.*, 1993).

It goes without saying that this type of "heavy" endoscopy (performed, however, on an out-patient basis) is directed toward high-risk patients who have, or are suspected of having, aerodigestive tract cancer. At the same time, in Europe it represents our main means of diagnosis of curable esophageal cancer.

### Endoscopic Morphology

Any classification of the endoscopic morphology of early esophageal carcinoma must include the description of the fundamental lesions as well as of their mapping (Table 2).

Most often, the fundamental lesions are characterized by both a color change (leucoplakia or erythroplakia) and a surface alteration (elevated or depressed). There exists, moreover, a relationship between these two morphologic characteristics: an elevated lesion is generally associated with a leucoplakia, whereas a depressed lesion usually shows up as a reddish lesion at endoscopy (erythroplakia). However, an esophageal carcinoma in situ can be flat (no surface alteration) and show no color change; this is called

**Table 2** Fundamental lesions and mapping of early esophageal carcinomas

| | | |
|---|---|---|
| Fundamental lesion | color change | white |
| | | red |
| | | mixed (red + white) |
| | | no change |
| | surface irregularity | elevated |
| | | flat |
| | | depressed |
| Mapping | single lesion | |
| | multicentric lesion | |

Note.—The combination of the fundamental lesions and of their mapping results in the classification of four main types of early esophageal carcinomas.

the "invisible" form (or "occult" according to the Chinese) of early esophageal cancer.

After the combined study of the morphologic aspects of the fundamental lesions and their mapping, we classify early esophageal carcinomas into four types (Siewert *et al.*, 1977).

### Leucoplakia

The distinctive morphologic element is the localized and elevated surface alteration, associated with a whitish discoloration. Usually, the lesion is unique and quite distinct from the surrounding normal mucosa. Toluidine blue staining is of minimal help in this type, because the surface keratosis keeps the dye from penetrating into the mucosa. The superficial extension generally is moderate, and multicentricity is absent or minimal. On the other hand, intraluminal growth and deep infiltration of the organ wall are common, especially if this plakelike lesion originates from severe dysplasia of the basal layers of the epithelium with early infiltration of the basal cell membrane and with an intact superficial layer of the epithelium. For this reason, endoscopic distinction between a carcinoma in situ and a cancer that has already infiltrated the submucosa is not easy. The slightest rigidity of the wall is a sign of submucosal invasion. (Fig. 1).

### Erythroplakia

This is the most common type, although often it is barely visible. The dominant feature of this lesion is the reddish discoloration, which is in general diffuse and slightly depressed compared with the surrounding normal mucosa. Given the multicentricity and diffuse nature of the lesion, it is impossible to determine the exact extent of the lesion without the use of vital staining. In these cases, use of Toluidine blue as a tumor marker is of particular help in determining the precise limits of the tumor, which often is multicentric, and for directing biopsy. These lesions are

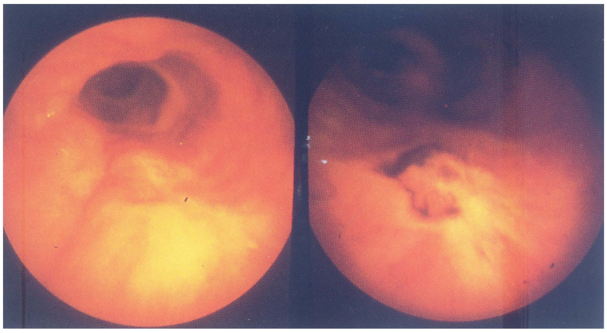

**Figure 1a, b** Plakelike form of early esophageal cancer with hardly any surface alteration. a) Although this invasive form of early cancer hardly shows any surface discoloration, the plakelike rigidity of the mucosa is suspicious for a submucosal infiltration. b) After Toluidine blue staining, the lesion is somewhat better visualized, but the uptake of the dye is only partial.

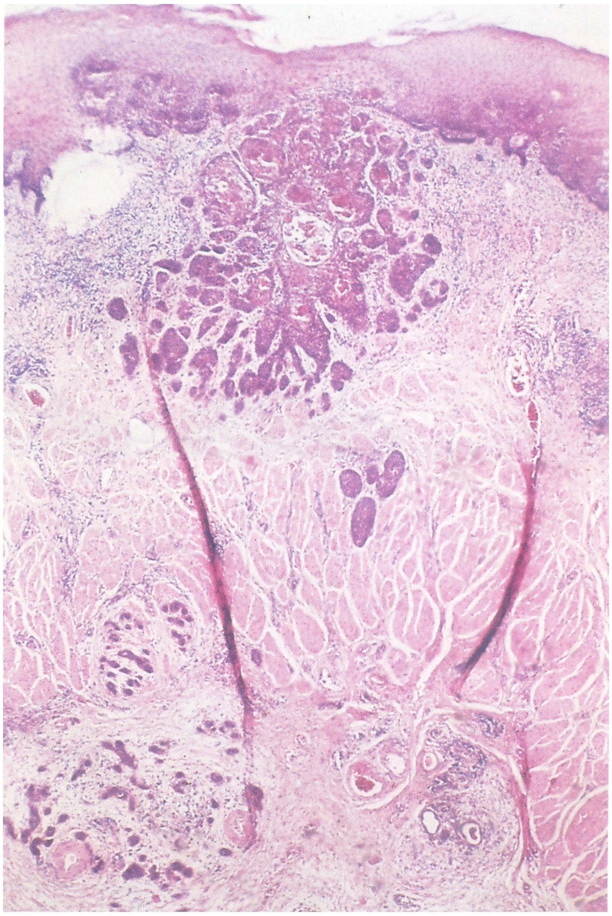

**Figure 1c** Histologic section showing a submucosal extension, whereas the superficial layers of the epithelium still remain intact. This type of lesion is derived from a severe dysplasia of the basal layers of the squamous cell epithelium. Invasion of the lamina propria and submucosa may occur before any significant surface alteration is visible. This probably corresponds to a most aggressive type of early carcinoma.

characterized by their superficial spread, which is often extensive (several cm²), whereas intraluminal and intraparietal growth appear only later.

It is important to note that a perfectly flat erythroplakia can correspond to multiple microinvasive cancer foci. The multicentricity of the lesion exists not only at the stages of dysplasia and carcinoma in situ but also in the invasive stage (Fig. 2).

### Leucoerythroplakia

This is the mixed type, composed of white, localized, elevated lesions, which are quite distinct and diffuse disseminated erythroplastic lesions. After Toluidine blue staining, the elevated easily visualized whitish lesions color very slightly, whereas the poorly visualized reddish lesions turn royal blue. The leucoerythroplakia tends to correspond to the most advanced form of early squamous cell carcinoma in the esophagus, especially if the superficial extension is greater than 2 cm² (Fig. 3).

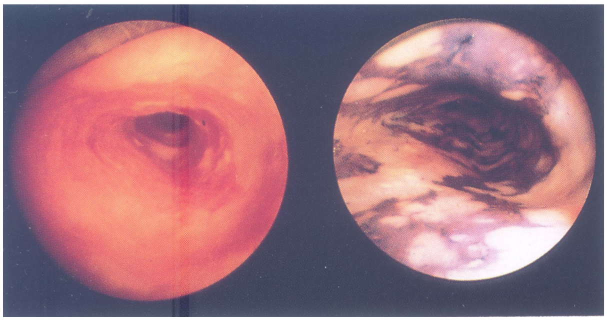

**Figure 2a, b** Microinvasive carcinoma of the esophagus showing as a circular erythroplakia over several square centimeters. a) The endoscopic aspect without vital staining is difficult to differentiate from a Barrett's esophagus, for example. b) After vital staining with Toluidine blue, the lesion shows as multicentric foci of royal blue areas intermingled with normal unstained mucosa.

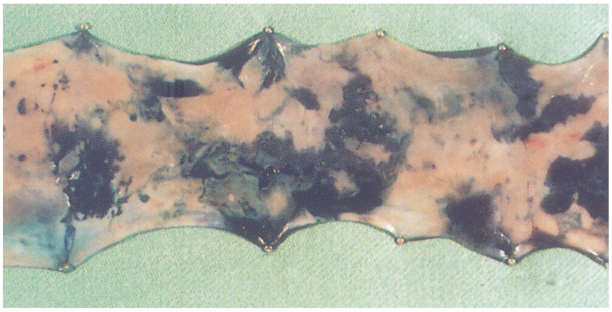

**Figure 2c** On the resected specimen colored with Toluidine blue, the lesion extends circumferentially over several centimeters in length.

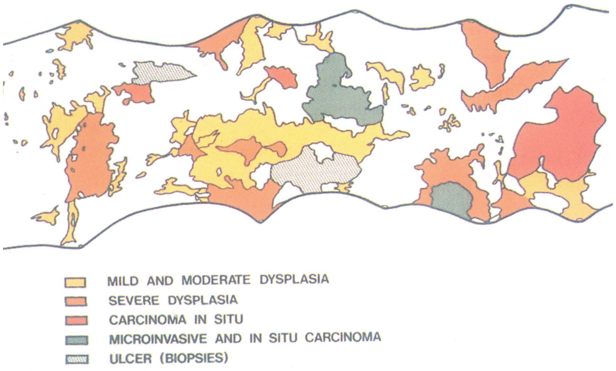

**Figure 2d** Histologic mapping of the lesion. In spite of a very large superficial extension (several square centimeters and circumferential), the lesion did not invade beyond the muscularis mucosae, showing that an extensive but flat and nongranular erythroplakia may still correspond to a very early lesion.

### Occult Form

This is the "invisible" form of dysplasia and carcinoma in situ. In our series, it never corresponded to an invasive early carcinoma. This occult form is detected only by vital staining techniques (Toluidine blue, Lugol Iodine solution). It probably corresponds to a forerunner of the other types, mainly the erythroplakia. Often these lesions are multicentric right from the start (Fig. 4).

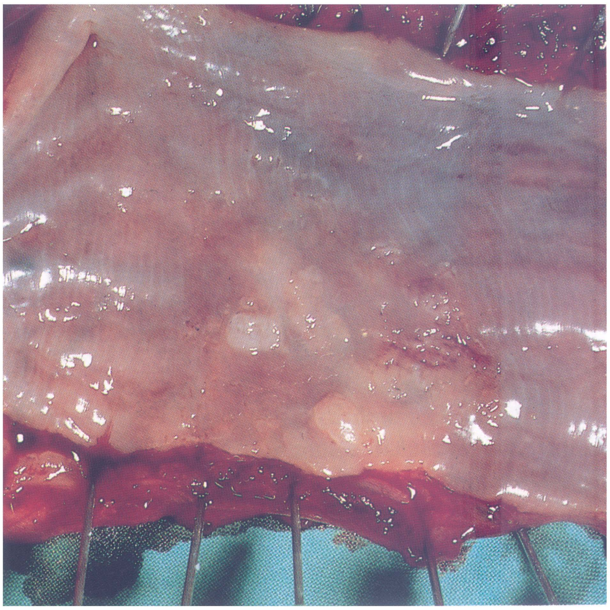

**Figure 3a** Resected specimen of a leuco-erythroplastic lesion showing as microinvasive carcinoma at histology analysis. a) The lesion shows as multicentric foci of erythroplakia, intermingled with white, elevated, and well-circumscribed leucoplakias.

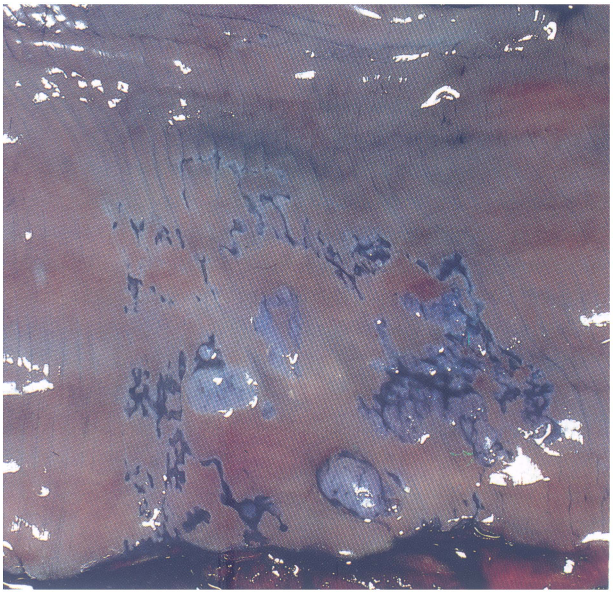

**Figure 3b** After Toluidine blue staining, leucoplakias stain very little, whereas erythroplakias turn a royal blue. The precise delineation of lesions is evident after Toluidine blue.

### Histology Method

Given the approximately 80% rate of multicentric tumors and the fact that it is impossible to microscopically determine the zones of maximal invasion, a histopathologic examination of step sections of the surgical specimen is necessary in all cases of early cancer.

The specimen is colored using 1% Toluidine blue solution immediately after surgical removal in the operating room. The stain shows less affinity for poorly vascularized tissue, and too long a delay between the time of specimen excision and vital staining can result in false-negative results.

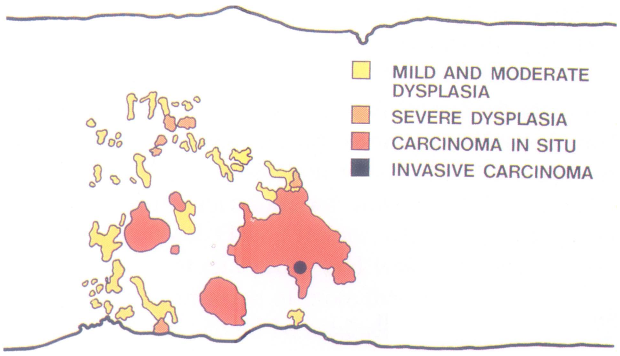

**Figure 3c** Serial histologic sections are necessary to establish a precise histologic mapping. Here, multiple foci of dysplasias and carcinoma in situ are evident with a single spot of intramucosal invasion.

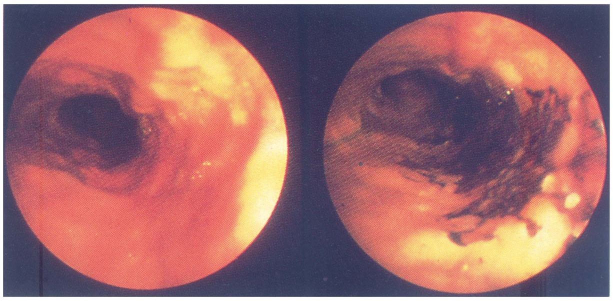

**Figure 4a, b** Occult form of early esophageal carcinoma. a) Before vital staining with Toluidine blue, this carcinoma in situ of the posterolateral wall of the esophagus is invisible at endoscopy. b) The Toluidine blue stain reveals the multicentric aspect of the lesion. The delineation is precise with a longitudinal extension of 5 cm over one third of the circumference of the esophagus.

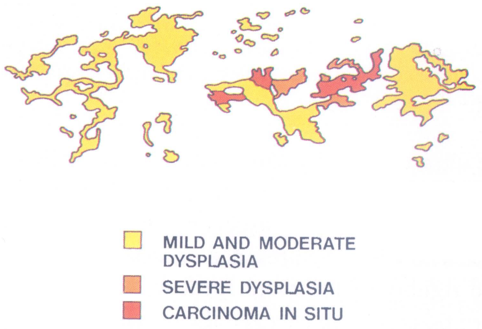

**Figure 4c** The mapping of the resected specimen shows multiple foci of dysplasias of all grades and of carcinoma in situ. Despite a superficial spread of several square centimeters, this occult lesions shows no sign of microinvasion on serial histologic sections (around 80 histological slides).

The specimen is then pinned to a cork board and photographed to life size (format 1/1). After formalin fixation, all lesions are examined histologically by step sections (each 2 mm). The anatomic site of the sample immediately is labelled on a life-size photograph and is precisely oriented by coloring its proximal end with India ink. This provides precise evaluation of the histologic slide and an exact reconstitution of the mapping of the tumor (Fig. 5).

Another method of anatomopathologic examination was proposed by Mandard et al., in 1980 (Mandard *et al.*, 1980). It consists of painting all macroscopically visible lesions as well as those discovered by vital staining with green China ink; this makes all the pathologic zones immediately obvious on the histologic slides.

### Staging of Early Esophageal Cancers

The choice of an optimal treatment modality depends on an exact endoscopic staging of the lesion. Beside endo-

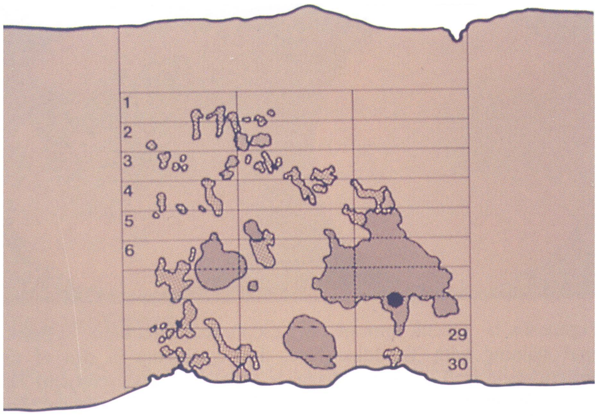

**Figure 5** Method of histologic examination of surgical specimens. 1) Vital staining with Toluidine blue in the operating room, immediately after removal. 2) Optimal spreading of the specimen on a cork board and fixation in formalin. 3) Polaroid photograph 1/1 for precise annotation of the histologic sampling 4) Step sections (an average of 5 $cm^2$) of all microscopically visible lesions and those revealed by Toluidine blue staining. 5) Orientation of each sample by coloring the proximal extremity with black China ink.

scopic ultrasonography, three main criteria help differentiate in situ, microinvasive, and submucosal carcinomas. These are the endoscopic aspect of the lesion, its superficial extension, and the presence or absence of a slight localized rigidity of the esophageal wall. In a prospective study of 64 cases, we checked the reliability of our endoscopic criteria in predicting the in-depth invasion of early squamous cell carcinomas of the aerodigestive tract (Monnier *et al.*, 1991). On the basis of these criteria, the endoscopist establishes the presumed histologic diagnosis of the lesion. Using a secret code, he stages the lesion as an intraepithelial carcinoma, as a microinvasive carcinoma not invading beyond the muscularis mucosae, or as a submucosal carcinoma.

The presumed diagnosis is passed on to the pathologist using the secret code number. The resected specimen is analyzed by serial histologic sections, and the degree of cancer invasion is measured by micrometry. The histologic diagnosis then is confronted to the endoscopic diagnosis by a third person who establishes a correlation between the two.

### RESULTS

Knowledge of the different morphologic aspects of early esophageal carcinomas plays a key role in determining the exact staging.

Among the macroscopic criteria that were studied, the color of the lesion, the superficial spread, and the parietal rigidity proved to be most useful in estimating the in-depth tumoral invasion during endoscopy.

### Morphological Aspects

The different fundamental lesions (occult form, erythroplakia, leucoplakia, and leucoerythroplakia) are not related to the same risk of tumor invasion (Table 3). The occult form is never invasive. In our series, this was confirmed in all 9 cases of early esophageal carcinomas. In another 18 cases, the same observation was made in the oral cavity and pharynx. These data are in full agreement with Chinese results on larger series of early esophageal cancers.

Pure erythroplakia is either intraepithelial or microinvasive (46 of 49 cases). Exceptionally, the in-depth invasion reaches the submucosa when the superficial extension is larger than 2 $cm^2$ or when slight rigidity of the wall, associated with some surface irregularity, is present. The leucoerythroplakia almost always corresponds to an invasive lesion, and a submucosal extension must be suspected in almost 40% of cases. The slightest rigidity of the wall and a superficial extension of more than 2 $cm^2$ are further important signs related to submucosal extension. In the esophagus, the leucoerythroplakia thus corresponds to the most advanced type of early cancer, according to our criteria.

Leucoplakia may correspond to an intraepithelial, intramucosal, or submucosal lesion. The degree of superficial extension is usually small. The presence of localized rigidity of the wall is the only sign that helps determine the submucosal infiltration when it is present.

A particular plakelike form of early esophageal cancer can be extremely difficult to diagnose, because it does not show any surface discoloration. Only a slight plakelike rigidity of the wall, without any surface alteration, is visible. This form of early esophageal cancer derives from dysplasia of the basal layers of the squamous cell epithelium, with early invasion of the lamina propria but without any dysplastic modification of the superficial layers of the epithelium. This gives rise to an aggressive and invasive squamous cell cancer of the esophagus.

Overall, the erythroplakias and occult forms represent 58% of all early esophageal cancers (Table 3). This indicates that the less visible lesions occur most frequently. This probably explains the poor diagnostic yield of routine upper GI endoscopies in detecting early esophageal cancers, at least in Western countries (Froelicher and Miller, 1985).

### Superficial Extension

When a lesion shows a superficial extension less than 1 $cm^2$, a microinvasion is likely in only 20% of the cases. This figure rises to almost 90% if the lesion is larger than 2 $cm^2$, whatever the fundamental lesion may be.

**Table 3** Morphology versus in-depth invasion in 100 early esophageal carcinomas

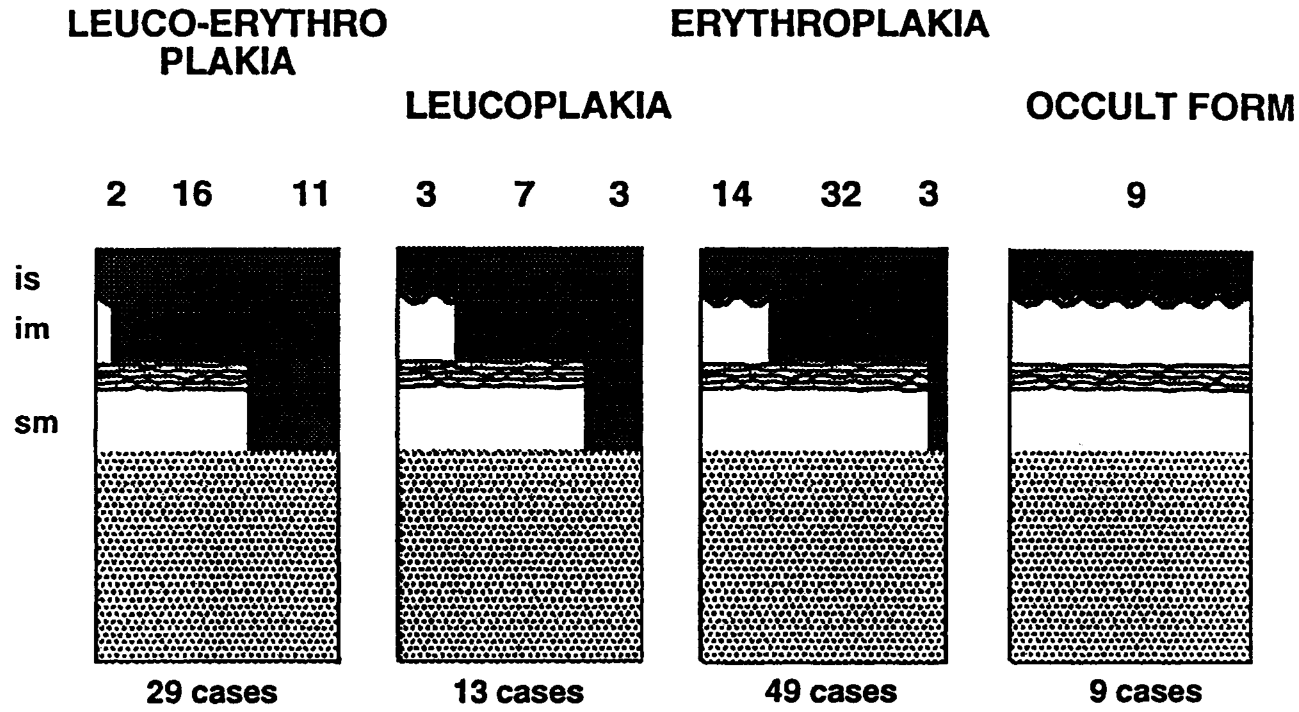

Note.—The erythroplakias and occult forms represent 58% of all early esophageal cancers. Endoscopically, they are the most difficult to diagnose because of their almost normal appearance with respect to the surrounding normal mucosa. They represent, however, the most relevant lesions to diagnose, because 55 of 58 correspond either to in situ or to intramucosal carcinomas, the prognosis of which is excellent. The leucoerythroplakia corresponds to the most advanced form of early carcinomas, because almost half of the cases already invade the submucosa. The pure leucoplakia, easily detected at endoscopy, is rare (13% of cases) and many correspond to in situ as well as to submucosal cancer.

### Rigidity of the Wall

This sign of invasion has been observed in 16 of 17 submucosal carcinomas in this series. The only case that was underestimated corresponded to an erythroplakia with a superficial extension of more than 2 $cm^2$. It was initially staged as an intramucosal carcinoma, because no real rigidity of the wall could be determined at endoscopy.

Combining these three criteria, we were able to predict accurately or overevaluate the degree of in-depth invasion in 55 (86%) of 64 early cancers of the aerodigestive tract that were studied prospectively as described in *Material and Methods*. In 9 (14%) cases, the lesion was underevaluated at endoscopy, but on each occasion the underestimation was minimal. Only 3 lesions, which were considered microinvasive at endoscopy and which eventually corresponded to an invasion of more than 1 mm (twice 2 mm and once 3 mm) could be assumed to have real negative therapeutic consequences, because the submucosal extension bears a higher risk of lymph node metastasis than does an intramucosal invasion. It is interesting that the 3 underevaluated lesions corresponded to a leucoerythroplakia in 2 cases and to an erythroplakia in 1 case, which confirms their high tendency toward cancerous infiltration.

### DISCUSSION

### Definition of Early Squamous Cell Carcinoma of the Esophagus

At present, the definition of early esophageal carcinoma is based on the empirical notion of surgical curability. Anatomically, it concerns carcinomas limited to the mucosa and submucosa without lymph node metastasis ($pT_1aN_0$ and $pT_1bN_0$). It is debatable, however, if submucosal carcinomas still should be considered as early cancers. First, around 70% of the cases initially present with lymph node metastasis at surgery, and second, $T_1bN_0$ cancers may develop late lymph node metastasis, as we ourselves have documented in 11 consecutive cases followed up for more than 5 years. None of these 11 cases showed evidence of second primaries in the lungs, esophagus, or upper aerodigestive tract at autopsy. This means that submucosal carcinomas of the esophagus, even without lymph node metastasis at the time of surgery, are far from having 100% cure rates. We therefore propose that the definition of early esophageal cancer should be restricted to in situ and intramucosal carcinomas without lymph node metastasis ($pT_1aN_0$).

### Pretherapy Staging of Early Esophageal Cancer

This includes the precise evaluation of the in-depth invasion of the primary tumor and the detection of mediastinal lymph node metastasis. Furthermore, at least in Europe where the incidence of esophageal carcinoma is related to tobacco and alcohol consumption, a routine pretherapy bronchoesophagoscopy should be performed in all cases to detect synchronous early asymptomatic cancers of the oral cavity, pharynx, or tracheobronchial tree. This is especially worthwhile in former head and neck cancer patients.

As we have demonstrated above, the endoscopic staging of esophageal cancers is based on the morphology, surface extension, and slight parietal rigidity of the lesion. This allows one to predict correctly the degree of in-depth invasion in approximately 95% of cases. Only 3 of 64 lesions were underevaluated in our series and could thus have been responsible for a negative therapeutic consequence. This means that a well-trained endoscopist can differentiate an intramucosal from a submucosal carcinoma with a fairly high degree of accuracy.

As far as the detection of mediastinal lymph node metastasis is concerned, only endoscopic ultrasonography (EUS) really is useful. Of 113 squamous cell carcinomas, Tio *et al.*, (1989) demonstrated a positive correlation between EUS and histology in 100% of $T_1$ and $T_2$ esophageal cancers, whereas the rate was lower for $T_3$ and $T_4$ cancers. In the latter, the tumoral stenosis often prevents the progression of the EUS device beyond the tumor to examine the lymph nodes.

Overall, the accuracy of the pretreatment staging (tumor and lymph nodes) is approximately 82% with the EUS for $T_1$ and $T_2$ cancers. This figure drops to only 12% with CT. In view of these data, it seems that our endoscopic staging system, combined with the endoscopic ultrasonography for the detection of lymph node metastases, represents the best pretherapy investigation to predict the future success of a pure endoscopic treatment of early esophageal carcinomas. EUS may further reinforce or invalidate the endoscopic staging of the primary tumor based on endoscopic criteria.

### Treatment of Early Esophageal Cancer

In 1980, the early report of Earlam and Cunha-Melo (1980) on the surgical treatment of esophageal carcinoma mentioned a resectability rate of only 22% and a poor 5-year survival rate of only 3%.

Because of the greater availability of endoscopy, allowing diagnosis at an earlier stage, and of the expertise of some surgical centers, the prognosis and outcome of this dismal disease have steadily improved over the last decade. More radical resection procedures with posterior mediastinectomy (Akiyama *et al.*, 1981; Lerut *et al.*, 1992), en bloc resection (Altorki and Skinner, 1990), and three field dissections (Tsurumaru *et al.*, 1990) have been developed to increase the chance for local control and cure.

If 5-year survival rates remain around 90% in stage I cancers (Lerut *et al.*, 1992), they are deceptively low for stage III and to a lesser extent for stage II lesions, at least in Western countries where tobacco and alcohol consumption plays a major role as risk factors. The debilitated patients, often presenting with multiple internal pathologies, represent poor surgical candidates.

Although some specialized centers in thoracic surgery report a resectability rate of 77% (Lerut *et al.*, 1992) for all esophageal cancers, one must admit that the largest group of patients are detected at a late stage of the disease (stages III and IV). The only hope for cure, therefore, lies in the earlier detection of esophageal carcinomas. As long as mediastinal lymph nodes are free of metastasis at surgery, superficial cancers have a good prognosis. Several groups from Japan (Endo *et al.*, 1986; Japanese Research Society for Esophageal Disease, 1989), China (Chinese Academy of Medical Science and Honan Province, 1973) and Western countries (Lerut *et al.*, 1992; Altorki and Skinner, 1990; Gignoux and Segal, 1984) have shown that the 5-year survival rates of $pT_1N_0$ carcinomas have increased from 60% to almost 90%, depending on the proportion of $T_1a$ and $T_1b$ carcinomas included in their series.

The safest treatment modality for early esophageal carcinoma is surgical resection with mediastinal lymph node dissection. There are, however, poor surgical candidates presenting with multiple associated pathologies, multiple synchronous or metachronous head and neck cancers, advanced age, and poor general condition caused by alcohol consumption, and other factors. Aggressive surgical treatment often is associated with a high morbidity and mortality in these patients.

For obvious ethical reasons, an endoscopic treatment of early esophageal carcinoma can be foreseen only after a thorough collegial discussion including surgeons, radiotherapists, chemotherapists, and the patient. It is clear that a final decision can be taken only according to each case, given the complexity of the parameters affecting each patient (age, general stage, associated illness, previous oncological treatments and synchronous multiple cancers).

At present, the endoscopic cure of early cancers can be approached in three different ways:

1. *Endoscopic mucosectomy.* This technique, described by Inoue and Endo (1990), is applicable to small lesions only, with a limited superficial spread. It has the great advantage of providing a surgical specimen for a histologic examination. Technically, it is not always easy to perform and cannot be used for large superficial extensions.
2. *Vaporization with a thermal laser.* This technique is only applicable to small lesions, the superficial extension of which is about 1 $cm^2$. A carbon dioxide laser is preferable to a Nd:YAG laser for better control the in-depth vaporization without risk of inducing a secondary perforation of the organ wall.
3. *Photodynamic therapy.* In the esophagus, this treatment modality allows one to cure widespread superficial lesions (several $cm^2$) provided they remain intramucosal ($T_1aN_0$) and that they are not circumferential. A circular mucosal necrosis would induce a stenosis during the healing process. Our experience with PDT of 35 early esophageal carcinomas (Monnier *et al.*, 1992) has shown that the results are quite favorable for in situ and intramucosal carcinomas, with a recurrence rate of less than 8% (2/27 lesions). For submucosal carcinomas, the local recurrence rate is much higher (~25%), and the risk of lymph node metastases must not be underestimated.

In a good surgical candidate, a $T_1bN_0$ carcinoma must be treated surgically, with a complete esophageal resection and an extensive mediastinal lymph node dissection.

### CONCLUSIONS

Given our present knowledge, early esophageal carcinomas can be characterised as follows:

1. The definition of early esophageal carcinoma should be restricted to $pT_{1a}N_0$ cancers.
2. The endoscopic aspect of in situ and microinvasive carcinomas is variable, and their early diagnosis by endoscopy is difficult. They are classified into four morphologic types.
3. The most common endoscopic aspect is the erythroplakia, flat or slightly depressed, often multicentric (80% of cases).
4. Toluidine blue or the Lugol iodine solution are of considerable help in determining the precise topography and extent of early cancers, which are poorly visible or invisible (erythroplakia and occult forms).
5. Based on morphologic criteria, superficial spread, and localized rigidity of the wall, it is possible to estimate the endoscopic staging of an early carcinoma with a fairly high degree of accuracy (approximately 95%).
6. EUS is invaluable in the staging of lymph node metastases and may help confirm or invalidate the stage of in-depth tumoral invasion.
7. In poor surgical candidates, photodynamic therapy or endoscopic mucosectomy represents the best treatment options for $T_{1a}N_0$ carcinomas.
8. For $T_{1b}N_0$ carcinomas, an esophageal resection with extensive mediastinal lymph node dissection must be undertaken in good surgical candidates. In inoperable patients, PDT combined with radiotherapy represents an alternative treatment.
9. For epidemiologic reasons (alcohol and tobacco consumption in Western countries), the pretherapy workup of early esophageal cancers should include a bronchoesophagoscopy with a complete otolaryngologic examination to detect early synchronous cancers of the mouth, pharynx, or tracheobronchial tree.

### REFERENCES

Akiyama H., Tsurumaru M., Kawanura T., *et al.* (1981) Principles of surgical treatment for carcinoma of the esophagus: analysis of lymphnode involvment. *Ann Surg*, 194:435–446.

Altorki N. K., Skinner D. B. (1990) En bloc esophagectomy: the first 100 patients. *Hepatogastroenterology*, 37:360–363.

Coordinating Group for Research on Esophageal Carcinoma. Chinese Academy of Medical Science and Honan Province: The early detection of carcinoma of the esophagus. *Scientia Sinica*, (1973)16:458–463.

Earlam R., Cunha-Melo J. R. (1980) Oesophageal squamous cell carcinoma I: a critical review of surgery. *Br J Surg*, 67:381–390.

Endo M., Takeshita K., Yoshibo M. (1986) How can we diagnose the early stage of esophageal cancer? Endoscopic diagnosis. *Endoscopy*, 18:11–18.

Froelicher P., Miller G. (1985) Esophageal cancer limited to the mucosa and submucosa in Europe. In *Esophageal Disorders: Pathophysiology and Therapy*. Raven Press, 355–357.

Gignoux M., Segal P. (1984) *Les Cancers de L'oesophage en 1984, 135 Questions*. Maloine, Paris, 318–321.

Inoue H., Endo M. (1990) Endoscopic esophageal mucosal resection using a transparent tube. *Surg Endoscop*, 4:198–201.

Lerut T., DeLeyn P., Coosemans W. *et al.* (1992) Surgical strategies in esophageal carcinoma with emphasis on radical lymphadenectomy. *Ann Surg*, 216:583–590.

Mandard A. M., Tourneux J., Gignoux M. *et al.* (1980) In situ carcinoma of the esophagus: macroscopic study with particular reference to the Lugol test. *Endoscopy*, 12:51–57.

Monnier Ph., Fontolliet Ch., Wagnières G. (1992) Further appraisal of PDI and PDT of early squamous cell carcinomas of the pharynx, oesophagus and bronchi. In: *Photodynamic Therapy and Biomedical Lasers* Elsevier Science Publishers B.V., 7–14.

Monnier Ph., Pasche P. H., Fontolliet Ch. (1991) Staging endoscopique du carcinome épidermoïde précoce pour la voie digestive supérieure. (The endoscopic staging of early squamous cell cancer of the upper digestive tract.) *Acta Endoscopica*, 21:693–705.

Monnier Ph., Savary M., Pasche R. (1981) Apport du Bleu de Toluidine en cancérologie bucco-pharyngo-oesophagienne. (Contribution of Toluidine Blue to bucco-pharyngo-oesophageal cancerology.) *Acta Endoscopica*, 11:299–315.

Monnier Ph., Savary M., Pasche R., *et al.* (1981) Intraepithelial carcinoma of the oesophagus: endoscopic morphology. *Endoscopy*, 13:185–191.

Mori M., Adachi Y., Matsushima T., *et al.* (1993) Lugol staining pattern and histology of esophageal lesions. *Am J Gastroenterol*, 88:701–705.

Siewert R., Lepsien G., Peiper H. J. (1977) Das Karzinom von Ösophagus und Kardia. *Internist*, 18:451–462.

The report of treatment results of esophageal carcinoma in Japan, 1979–1982. Japanese Research Society for Esophageal Diseases, National Center, Tokyo, 1989:

Tio T. L., Cohen P., Coene P. P. (1989) Endosonography and computed tomography of esophageal carcinoma. *Gastroenterology*, 96:1478–1486.

Tsurumaru M., Akiyama H., Udagawa H. (1990) *Cervical-Thoracic-Abdominal Lymphnode Dissection for Intrathoracic Esophageal Carcinoma*. Futura Mount Kisco, New York, 187–196.

Tumor Prevention, Treatment and Research Group, Chengchow, Honan, China. (1977) Pathology of early oesophageal squamous cell carcinoma. *Chinese Med J*, 3:180–192.

